# Comparing encoding mechanisms in realistic virtual reality and conventional 2D laboratory settings: Event-related potentials in a repetition suppression paradigm

**DOI:** 10.3389/fpsyg.2023.1051938

**Published:** 2023-01-27

**Authors:** Marike Johnsdorf, Joanna Kisker, Thomas Gruber, Benjamin Schöne

**Affiliations:** Experimental Psychology I, Institute of Psychology, Osnabrück University, Osnabrück, Germany

**Keywords:** repetition suppression, ERPs, EEG, virtual reality, encoding, memory

## Abstract

Although the human brain is adapted to function within three-dimensional environments, conventional laboratory research commonly investigates cognitive mechanisms in a reductionist approach using two-dimensional stimuli. However, findings regarding mnemonic processes indicate that realistic experiences in Virtual Reality (VR) are stored in richer and more intertwined engrams than those obtained from the conventional laboratory. Our study aimed to further investigate the generalizability of laboratory findings and to differentiate whether the processes underlying memory formation differ between VR and the conventional laboratory already in early encoding stages. Therefore, we investigated the Repetition Suppression (RS) effect as a correlate of the earliest instance of mnemonic processes under conventional laboratory conditions and in a realistic virtual environment. Analyses of event-related potentials (ERPs) indicate that the ERP deflections at several electrode clusters were lower in VR compared to the PC condition. These results indicate an optimized distribution of cognitive resources in realistic contexts. The typical RS effect was replicated under both conditions at most electrode clusters for a late time window. Additionally, a specific RS effect was found in VR at anterior electrodes for a later time window, indicating more extensive encoding processes in VR compared to the laboratory. Specifically, electrotomographic results (VARETA) indicate multimodal integration involving a broad cortical network and higher cognitive processes during the encoding of realistic objects. Our data suggest that object perception under realistic conditions, in contrast to the conventional laboratory, requires multisensory integration involving an interconnected functional system, facilitating the formation of intertwined memory traces in realistic environments.

## 1. Introduction

In the course of evolution, the brain has developed in a three-dimensional environment and is therefore adapted to it (Ogmen et al., 2020). Its cognitive and emotional mechanisms are attuned to work within a responsive environment governed by a fixed set of physical, perceptual and probability laws. In the same way, every day memories are encoded from multimodal, contextually embedded experiences.

However, such considerations play a rather subordinate role in the design of conventional laboratory experiments for practical and scientific theoretical reasons (see Parsons, 2015). For example, presenting real-life objects as two-dimensional pictorial stimuli on a computer screen allows for rigorous control of the stimuli’s features, but obscures their actual meaningfulness (Holler et al., 2020). Especially, physical and semantic information are altered when, e.g., a car is presented on an otherwise blank 2D screen. Features such as its actual size, weight, distance, and representation within the viewer’s egocentric reference frame lose significance. These differences from its real-life equivalent weigh even more considering that cortical object processing is not completely invariant to the object’s physical properties (Holler et al., 2020). Consequently, those stimuli only serve as reminders of real-life objects (Schöne et al., 2015) and are met with disparate motivational processes (Schöne et al., 2021a). Despite the epistemological advantages of this classical approach, it is unclear to what extent it is the most appropriate and yields meaningful results (see Chirico et al., 2018; Shamay-Tsoory and Mendelsohn, 2019; Snow and Culham, 2021).

In line with this, a growing body of evidence suggests that memories of conventional laboratory events do not adequately reflect engrams formed under realistic, complex conditions (e.g., Cabeza et al., 2004; Cabeza and St Jacques, 2007). Rather, qualitative as well as quantitative differences are found in memories of conventional laboratory experiences and more naturalistic conditions (Cabeza et al., 2004; Cabeza and St Jacques, 2007). In particular, Virtual Reality (VR) experiences have been reported to facilitate the formation of profound memory traces functionally associated with autobiographical memory (AB, Kisker et al., 2021a,b; Schöne et al., 2019, 2021a). Albeit definitions of AB are up to discussion (e.g., Schöne et al., 2019), there is a consensus that AB comprises highly personal and self-relevant engrams (Conway, 2005; Cabeza and St Jacques, 2007; Schöne et al., 2018). VR seems to facilitate this self-relevant or egocentric moderated cognitive processing (see Schöne et al., 2019; Kisker et al., 2021b), but it is yet unclear whether this occurs at early stages of memory formation, the encoding.

As a correlate of successful encoding, Repetition Suppression (RS) could yield further insight into the formation of memory traces at early stages. In the RS paradigm, objects are presented twice. The associated RS effect is a well-established, priming-related decrease in neuronal activity, characterized by a sharper cortical stimulus representation in response to a second presentation (Desimone, 1996; Wiggs and Martin, 1998). In canonical studies on event-related potentials (ERP), analyzing the P1, N1, and later time windows, RS occurs at these later ERP complexes (e.g., Gruber and Müller, 2002, 2006; Gruber et al., 2004). It is hypothesized to reflect earliest instances of encoding processes and memory trace formation as it predicts memory performance, which is positively related to the magnitude of the RS effect (Sommer et al., 2021).

To address the aforementioned limitations of conventional 2D paradigms, the study at hand compares the temporal dynamics of object perception and subsequent encoding processes under realistic 3D and under conventional 2D conditions. We translated a classical RS paradigm to VR, thereby enhancing ecological validity (for review see Parsons, 2015; Smith, 2019).

The VR setup’s goal was to investigate how a deviation from the standard paradigm toward higher realism affects attentional and encoding processes, directly comparing the electrophysiological signals obtained under both conditions. If only minor perceptual differences were manifested in the results, this would suggest that the general conventions governing experimental psychology could be implemented without notable exception, since they would be replicable under such different conditions and modalities. Those minor perceptual differences comprise mechanisms such as allocating more attentional resources for virtual as opposed to planar monitor objects, compensating for the increased presentation size (for review see Wedel and Pieters, 2007). Hence, a stronger cortical response does not reflect a shift in the mode of operation *per se*. As a consequence, VR would not yield further insights into cognitive and emotional processing beyond the standard laboratory setup.

Yet our previous EEG-study investigating recognition memory for scenes provides evidence for a shift in the mode of operation regarding mnemonic retrieval processes (Kisker et al., 2021b). Further results suggesting divergent functioning of cognitive processes under VR conditions would expand our knowledge about realistic functional properties of cognitive mechanisms.

In the present study, we expected to observe the typical RS effect in the conventional laboratory PC condition. Whether the typical RS effect could be replicated similarly in the VR condition or whether it would exhibit divergent characteristics would provide insights into the properties of forming a memory trace under realistic conditions.

## 2. Materials and methods

### 2.1. Participants

Twenty-nine participants were recruited from Osnabrück University *via* mailing list, student Facebook groups, and the Universities online bulletin board in exchange for partial course-credits or 15€. They neither did report any current or previous psychological, psychiatric or neurological disorder nor substance abuse. Three participants prematurely terminated the experiment due to technical problems and one participant was excluded due to unfulfilled participation criteria, therefore data of 25 participants were included in analysis (7 male, 18 female, *M*_age_ = 21.68, *SD*_age_ = 2.91), which is based on and exceeds the usual sample size of previous studies on the RS effect (e.g., Rugg et al., 1995; Gruber and Müller, 2002; Gruber et al., 2004; Stefanics et al., 2020). All participants had normal or with contact lenses corrected-to-normal vision. All participants gave informed written consent and the study was approved by the local ethics committee of Osnabrück University.

### 2.2. Stimuli

**Figure 1:**
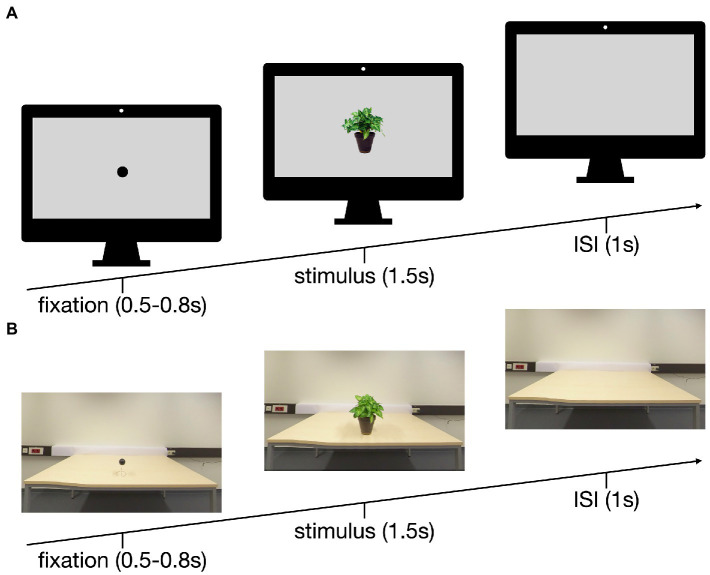
Setup of the trials in the PC **(A)** and the VR **(B)** conditions: 0.5–0.8 s fixation, 1.5 s stimulus, 1 s inter stimulus interval (ISI).

Three-hundred-sixty pictures of everyday objects were compiled into a databank (see Supplementary material S1). For the conventional laboratory condition, the pictures were stripped of their context, scaled to a standardized size and superimposed on a gray background (see Figure 1A). The VR pictures were taken with an Insta360 Pro 3D/360° camera at a resolution of 4 K (3,840 × 2,160 pixels). To this end, the stimuli were placed on a neutral table in front of a white wall (see Figure 1B). The height of the camera lenses was standardized for the average sitting human at 112 cm, the table height was 72 cm. The distance to the object was 62 cm, the object was placed 15 cm from the table edge resulting in a viewing angle of 34.2°, with the object maintaining it actual size. Preliminary VR tests with five subjects of different height (158 cm – 186 cm) and gender additionally confirmed the feasibility of this setup as they did not report any feeling of dysmorphia and could identify all objects in VR.

### 2.3. Procedure

Before the experiment, participants were interviewed regarding their psychological and medical history to assess their eligibility for the study. Following, the EEG was set up. Participants started counterbalanced either with the conventional laboratory condition or the VR condition. In each condition they were presented with 60 objects that were repeatedly presented and 60 that were presented only once. The repetition occurred with either one, two or three other objects (20 times each) in between the first and second presentation. The objects were drawn from the database in such way that they occurred either in the laboratory condition or the virtual condition but never in both. Objects were randomized across participants in such way that each object could randomly be presented either repeatedly or only once, either in the laboratory condition or in the VR condition. This algorithm makes object-specific effects unlikely.

For the conventional monitor setup, the participants were seated 115 cm from the monitor (24″, resolution of 1,902 × 1,200 pixels) resulting in a horizontal viewing angle of 5° and vertical viewing angle of 2.5° for the stimuli. Each of the 180 trials started with a dot fixation (black on gray; 500–800 ms), followed by an object (superimposed on a gray background, 1,500 ms) and a blank gray screen (1,000 ms; Figure 1A). The pictures were presented using Matlab (version R2021b, MathWorks, Natick, USA).

For the VR condition, an HTC Vive Cosmos^1^ was used and carefully placed over the EEG cap. The general layout and timing for the trials resembled the conventional laboratory setup. The dot fixation was a black sphere (diameter 6 cm) on a wire hovering 12 cm above the table. The fixation was replaced by an object, followed by a blank table (Figure 1B). Unity (version 2020.3.20f1, Unity Technologies, San Francisco, United States) was used for the VR environment.

### 2.4. EEG recording and analysis

An electroencephalogram (EEG) with 128 electrodes, attached in accordance with the international 10-20-system was recorded. Therefore, the requirement of 64 channels for a robust source analysis was met (Trujillo-Barreto et al., 2004). The Active-Two amplifier system from BioSemi (Amsterdam, Netherlands) was used with a sampling rate of 512 Hz and a bandwidth of 104 Hz (3 dB). A horizontal electrooculogram (hEOG) and a vertical electrooculogram (vEOG) were recorded, common mode sense (CMS) and a driven right leg (DRL) electrode were applied as ground electrodes. In the PC condition, the EEG was recorded with ActiView702 Lores (BioSemi, Amsterdam, Netherlands). In the VR condition, the EEG data stream and Unity triggers were recorded and synchronized using Lab Streaming Layer (LSL by SCCN^2^).

### 2.5. Preprocessing

The EEG data were preprocessed and analyzed with in-house Matlab-scripts implementing methods from EEGLAB v2021.1 (Delorme and Makeig, 2004). To this end, the data was referenced to average reference for further processing and filtered with an FIR band pass filter between 0.25 and 30 Hz. Flat (5 s no signal) and noisy channels (> four standard deviations for high-frequency noise) were removed and subsequently interpolated and detrended. Removal of artifacts (eye, muscle, heart, other noise) was conducted on basis of an ICA using the ICLabel function from EEGLAB. Stimuli-locked epochs had a duration of 500 ms before onset and 1,500 ms after onset. A baseline 300 ms before stimulus onset was subtracted before the grand averages were calculated. No trials were rejected from the reported results.

### 2.6. ERP components in electrode space: Data analysis

**Figure 2:**
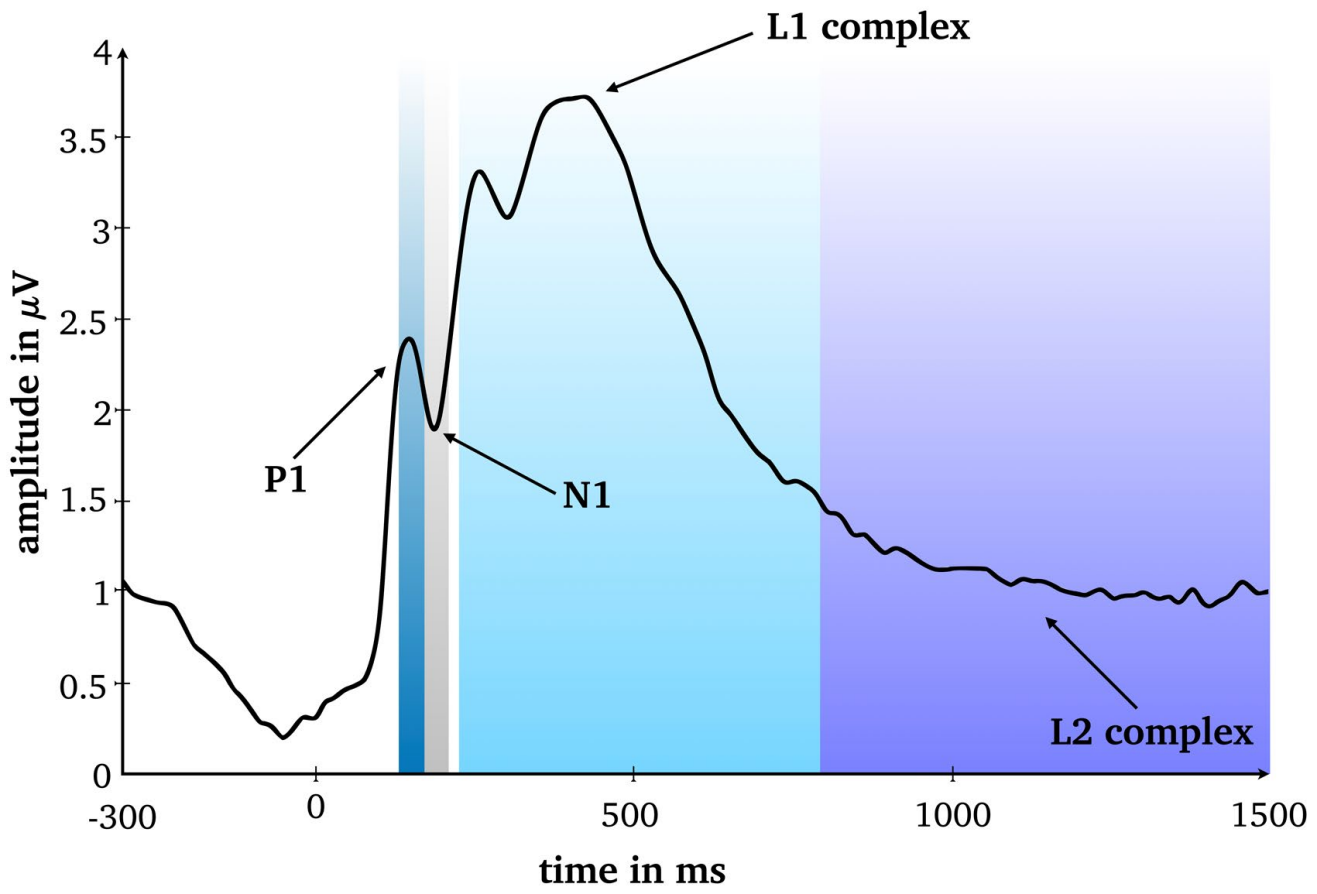
Root mean square averaged over conditions and all scalp electrodes. Four analyzed time windows are marked.

**Figure 3:**
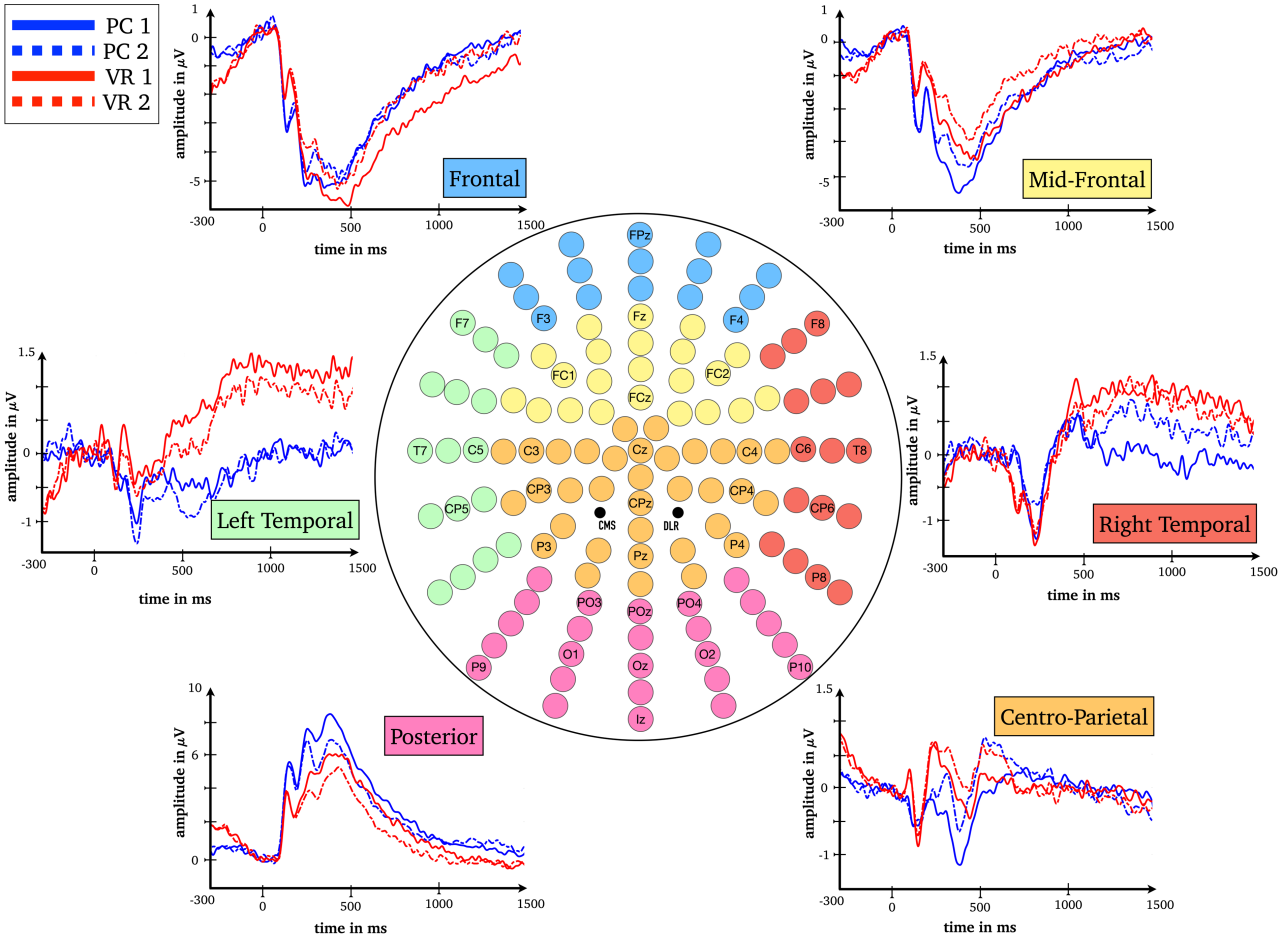
Baseline-corrected grand average waveforms (ERPs) for all combinations of the factors Modality (VR, PC), Repetition (first presentation, second presentation) and Cluster (frontal, mid-frontal, left temporal, right temporal, centro-parietal, posterior).

**Figure 4:**
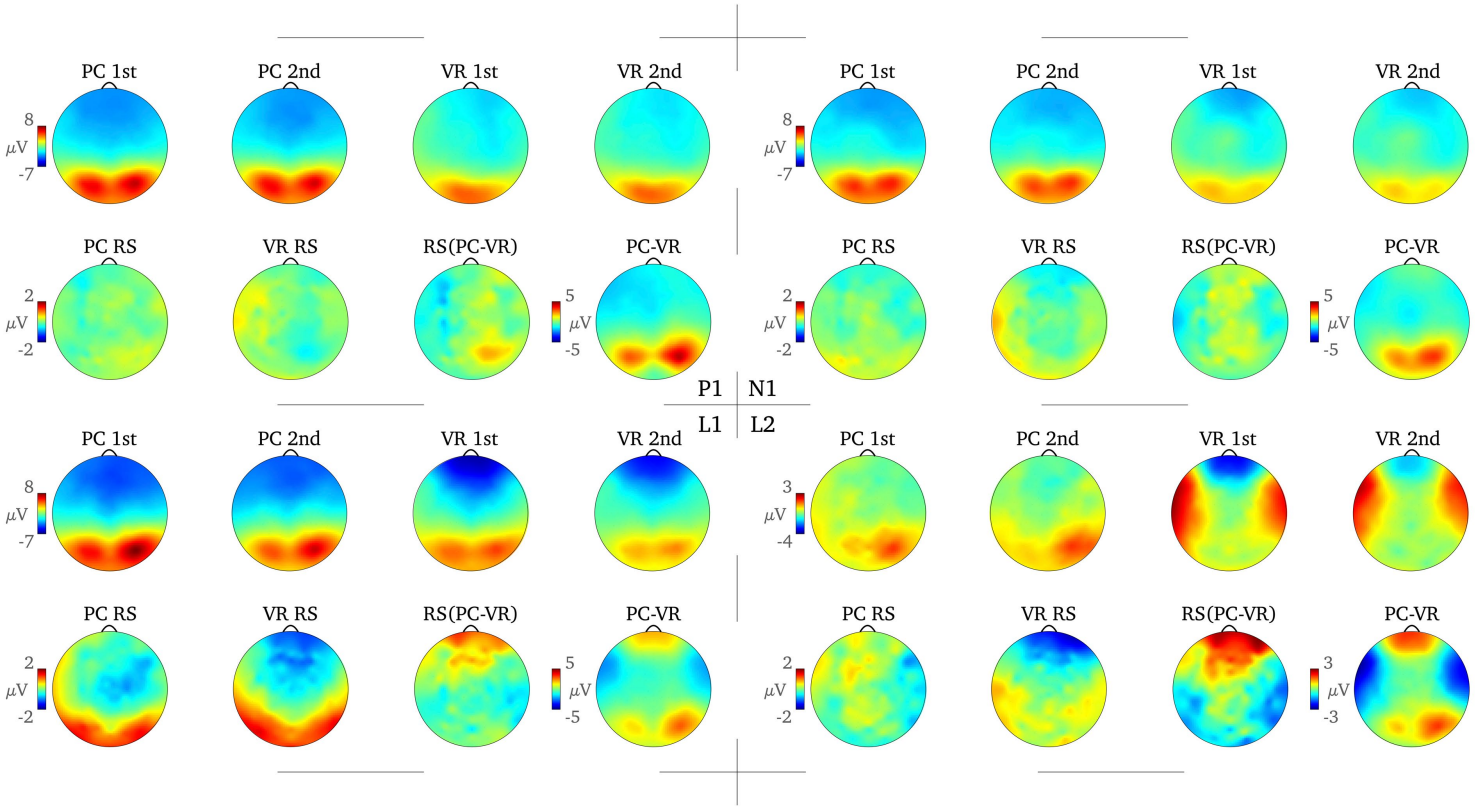
Topography of the amplitude separately for all combinations of the factors Modality (VR, PC) and Repetition (first presentation, second presentation) and the RS effect for each modality (PC RS, VR RS), the difference between the PC and VR RS effects [RS (PC-VR)] and the difference between the averaged amplitude in the PC and the VR condition (PC-VR) are displayed for each time window. The color scale bars on the left each refer to all following topographies.

To assess eligible time windows, the root-mean square averaged over conditions and all scalp electrodes was calculated. Based on visual inspection of the line plot, and in line with the scientific literature on RS under conventional laboratory conditions (Rugg et al., 1995; Gruber and Müller, 2002, 2006; Gruber et al., 2004) four time windows were analyzed for six typical electrodes clusters: P1 (125–165 ms), N1 (165–205ms), and two later complexes standard for RS research, the L1 complex (220–800 ms) and the L2 complex (800-1,500 ms), at frontal, mid-frontal, left temporal, right temporal, posterior and centro-parietal clusters (Figures 2–4).

The RS effects for the PC and VR conditions in each time window were calculated by subtracting the ERP amplitudes at the second presentation from those at the first presentation.

The statistical analysis was conducted in SPSS (Version 27). For each time window, a 2 × 2 × 6 repeated measurement ANOVA with factors *Modality* (VR, PC), *Repetition* (first presentation, second presentation) and *Cluster* (frontal, mid-frontal, left temporal, right temporal, posterior, centro-parietal) was calculated followed by corresponding post-hoc *t*-tests. For each time window, the post-hoc *t*-tests were calculated to compare the overall ERP amplitudes between the PC and VR conditions for each electrode cluster. For the time windows in which the RS effect occurred, the averaged amplitudes of the first and second presentation were compared for each electrode cluster. All reported ANOVA results are Greenhouse–Geisser corrected.

### 2.7. ERP components in source space

To examine the differences in activation of the cortical generators involved in visual perception and subsequent encoding of conventional and VR objects respectively, we modeled the sources of the effects of interests (see ERP components in electrode space) by means of a distributed source model variable resolution electromagnetic tomography (VARETA; Bosch-Bayard et al., 2001). VARETA computes the spatially smoothest estimates of the cortical generators and corresponds best to the amplitude distribution in the electrode space (Gruber et al., 2006b; Martens et al., 2011). The applied inverse solution comprised 3,244 grid points in a 3D grid as defined by a Leadfield matrix and corresponding to the placement of the 128-channel EEG (10-20-system).

**Figure 5:**
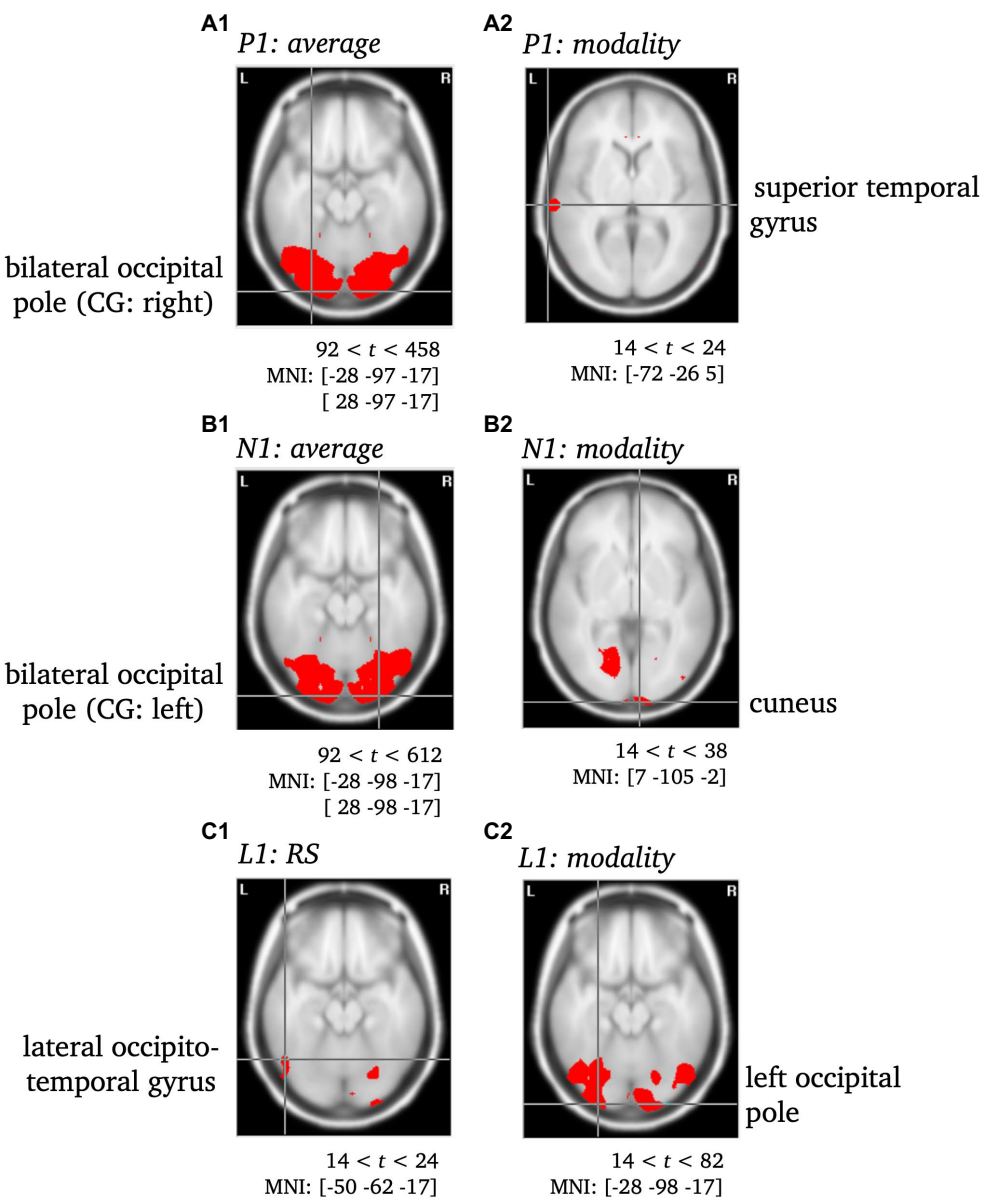
Statistically significant sources for the time span of the P1, N1 and L1. Statistically significant differences in activity are marked red, with *p* < 0.001. Per Panel, the center of gravity (CG) is labeled and the MNI coordinates [*x*, *y*, *z*] for the respective brain regions as identified using the Neuronic Tomographic viewer are given. See Supplementary Table S13 for further labels and MNI coordinates. CG, center of gravity.

**Figure 6:**
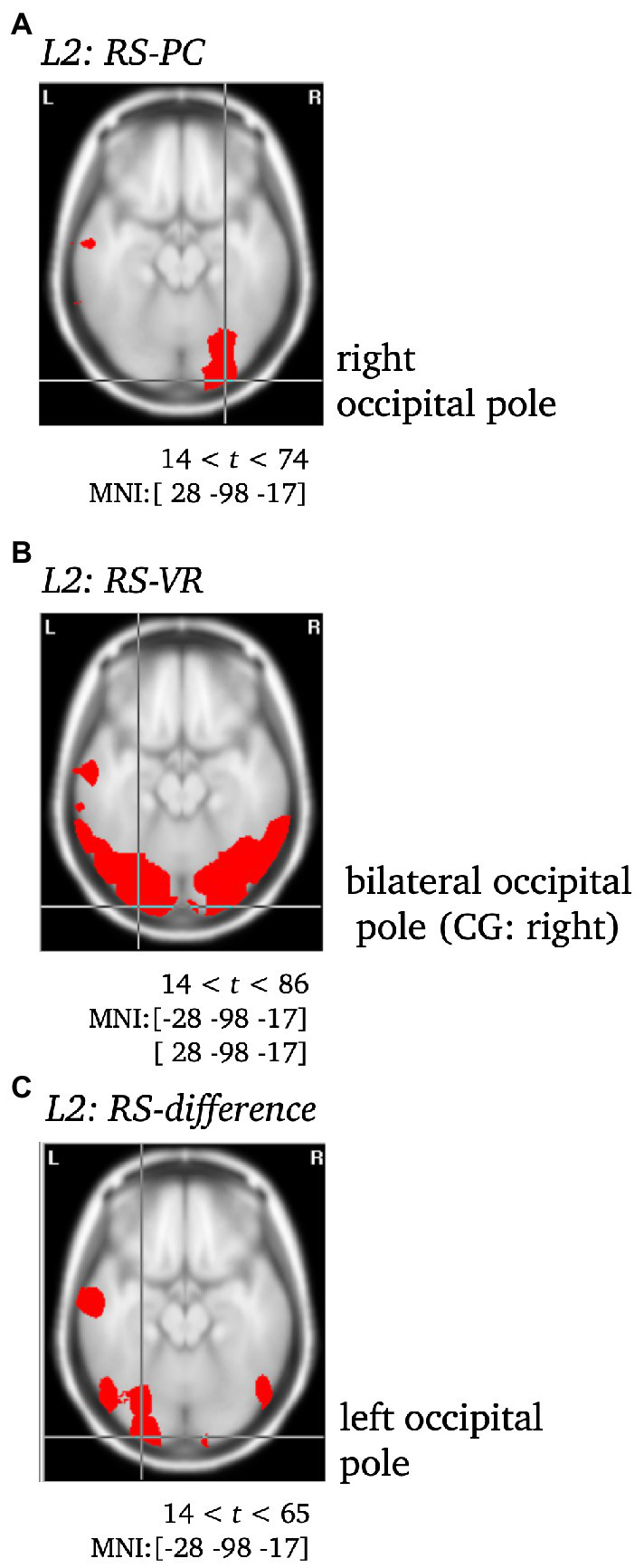
Statistically significant sources for the RS effect during L2 for PC **(A)**, VR **(B)** and the difference between both **(C)**. Statistically significant differences in activity are marked red, with *p* < 0.001. Per Panel, the center of gravity (CG) is labeled and the MNI coordinates [*x*,*y*,*z*] for the respective brain regions as identified using the Neuronic Tomographic viewer are given. See Supplementary Table S13 for further labels and MNI coordinates. CG, center of gravity.

To localize differences in activation patterns, *Hotellings T*^2^-test was performed per effect of interest with a significance level of *p* < 0.001. For all *t*-tests, the critical *t*-value was set using random field theory (Worsley et al., 1996, 2002). Accordingly, we used *t*_crit_ = 92 for testing the average across all conditions against zero (see P1, N1) and *t*_crit_ = 14 for all further comparisons between conditions (see Figures 5, 6). Significant voxels were projected onto the cortical surface constructed on the basis of the average probabilistic MRI brain atlas computed by the Montreal Neurological Institute (MNI; Evans et al., 1993). The brain region’s names for significant voxels were identified by the brain electrical tomography (BET) Neuronic Tomographic viewer.

**Figure 7:**
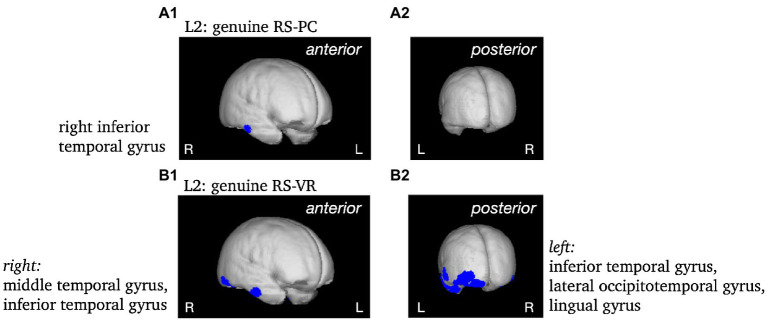
Non-statistical visualization of the genuine RS effect during L2 in the PC condition (panels **A**) and in the VR condition (panels **B**). MNI coordinates [*x*, *y*, *z*] for the respective brain regions as identified using the Neuronic Tomographic viewer: PC: right inferior temporal gyrus [57, −19, −24]; VR: right middle temporal gyrus [57,−11,−31], right inferior temporal gyrus [52, −63, −17], left inferior temporal gyrus [−43, −4, −39], left lateral occipitotemporal gyrus [−43, −84, −10], left lingual gyrus [−28, −84, −17].

To validate the use of VARETA with the current data set, the sources of the P1 and N1 components of the ERP were localized as a first step (see Figure 5). After a consistency check against previous publications (e.g., Di Russo et al., 2002; Gruber et al., 2006b), the sources of effects concerning further complexes of interest (L1, L2) were examined (see Figures 5, 6). Since the differences between conditions in the late L2 complex are of particular interest, the RS effect was visualized separately for VR and PC, with the genuine effect of each condition projected onto the cortical surface of a 3D brain (see Figure 7). In contrast to the previous Figures 5, 6, this representation is not based on the statistical parametric maps. For the visualization of the genuine VR effect, all regions that showed a PC effect were subtracted from the VR solution, as well as all regions that were not supported by the triple interaction Modality*Repetition*Cluster. Vice versa, for the genuine PC effect, all regions that showed a VR effect were subtracted, as well as all regions not supported by the aforementioned interaction.

## 3. Results

### 3.1. ERP components in electrode space

Due to the extensive statistical analysis, and to enhance readability, only the most important results are included in the text. Please refer to Supplementary Tables S2–S12 for the complete results.

For the P1 time window, the results show a main effect for the factors *Modality* [*F*_Modality_(1) = 6.74, *p* = 0.016; *η*^2^ = 0.22] and *Cluster* [*F*_Cluster_(1.31) = 50.42, *p* < 0.001; *η^2^* = 0.68] as well as an interaction between them (*F*_Modality*Cluster_(1.88) = 20.03, *p* < 0.001; *η^2^* = 0.45). Post-hoc tests reveal significant differences (all *p*s < 0.021; all *|ds| ≥ 0.496*) at all clusters between the VR and the PC condition (averaged over first and second presentation) except the centro-parietal cluster [*t*(24) = 0.58, *p* = 0.569; *d* = 0.116; see Figures 3, 4].

For the N1 window no main effect for *Modality* could be obtained [*F*_Modality_(1) = 2.64, *p* = 0.117; *η*^2^ = 0.10] but the main effect for *Cluster* [*F*_Cluster_(1.52) = 37.39, *p* < 0.001; *η*^2^ = 0.61] as well as the interaction between *Cluster* and *Modality* are highly significant [*F*_Modality*Cluster_(2.15) = 12.10, *p* < 0.001; *η*^2^ = 0.34]. Fewer clusters indicate functional differences in processing, namely the mid-frontal, the left temporal and the posterior clusters (*p*s < 0.044; *|ds|* ≥ 0.43). The frontal cluster reaches a trend toward significance [*t*(24) = −1.92, *p* = 0.066; *|d|* = 0.38], the right temporal [*t*(24) = 0.38, *p* = 0.705; *d = 0.08*] and the centro-parietal [*t*(24) = −0.83, *p* = 0.413; *|d|* = 0.17] clusters do not yield significant results, respectively (see Figures 3, 4).

In the L1 time window again an interaction between the factors *Modality* and *Cluster* reaches significance [*F*_Modality*Cluster_(1.63) = 7.02, *p* = 0.004; *η*^2^ = 0.23] and is accompanied by an interaction between factors *Repetition* and *Cluster* [*F*_Repetition*Cluster_(2.46) = 22.29, *p* < 0.001; *η*^2^ = 0.48]. The modality-independent RS effect reaches significance at all clusters (all *p*s ≤ 0.036; all *|ds|* ≥ 0.44), except for the right temporal cluster (*p* = 0.623; *|d|* = 0.10; see Figures 3, 4).

Finally, the L2 time window yielded the most important results as the three-way interaction is significant [*F*_Modality*Repetition*Cluster_(2.66) = 5.78, *p* = 0.002; *η*^2^ = 0.19]. The post-hoc *t-*tests reveal RS effects at frontal as well as midfrontal clusters only within the VR condition [*t*(24) = −3.85, *p* = 0.001; *|d| = 0.77* and *t*(24) = −2.17, *p* = 0.040; *|d|* = 0.43] but not in the PC condition [*t*(24) = 0.85, *p* = 0.406; *d* = 0.17 and *t*(24) = 1.42, *p* = 0.168; *d* = 0.28]. This effect is reversed at the right temporal cluster where there is only a RS effect in the PC condition [*t*(24) = −2.67, *p* = 0.013; *|d|* = 0.53], but not within the VR condition [*t*(24) = 0.99, *p* = 0.334; *d* = 0.20; see Figures 3, 4]. At frontal electrodes, the amplitude of the first presentation in VR was significantly more negative than the second presentation in VR [*t*(24) = −3.85, *p* = 0.001; *|d|* = 0.77] and compared to the amplitudes of the first and second presentations in the PC condition [*t*(24) = −2.45, *p* = 0.022; *|d|* = 0.49 and *t*(24) = −2.13, *p* = 0.044; *|d|* = 0.43; see Figures 3, 4]. To make sure that the RS effects within the conditions actually differed from the respective null effects in the other condition, the differences within the conditions were compared to each other [(PC 1^st^–PC 2^nd^) – (VR 1^st^–VR 2^nd^)]. As a result, the RS effects turned out to be specific for the respective condition as all tests were significant (all *p*s < 0.018; all *|ds|* ≥ 0.51).

### 3.2. ERP components in source space

*P1 & N1.* Both components were localized to the bilateral occipital pole, with the center of gravity either in the left (P1) or the right (N1) hemisphere when averaged across conditions and were thus consistent with previous literature (see, e.g., Di Russo et al., 2002; Gruber et al., 2006b). Moreover, the modality effect was characterized by significantly different activity of the superior temporal gyrus accompanying the P1 component and of the cuneus accompanying the N1 component (see Supplementary Table S13; Figure 5).

*L1*. The RS effect was localized to the left lateral occipitotemporal gyrus, whereas the modality effect was localized to the left occipital pole (see Supplementary Table S13; Figure 5).

*L2.* The VR RS effect yielded significantly different activity in the bilateral occipital pole (CG: right), but remarkably, in the middle temporal gyrus as well. In contrast, the PC RS effect was solely accompanied by significant differences in the activity of the right occipital pole. The difference between both was localized to the left occipital pole (CG), and yielded further significant differences in the left middle and the right inferior temporal gyrus (see Supplementary Table S13; Figures 6, 7).

## 4. Discussion

The present study aimed to investigate the formation of engrams for objects as its first instance under realistic conditions by comparing the classical Repetition Suppression (RS) paradigm between the conventional laboratory and realistic virtual environments. ERPs of four time windows for six typical electrode clusters were compared between the two experimental conditions to distinguish perceptual and mnemonic mechanisms. Overall, we observed lower ERP deflections, i.e., less negative or less positive ERP amplitudes, for most clusters of the P1 and several clusters of the N1, L1, and L2 in the VR as opposed to the PC condition. The typical RS effect was replicated in the PC condition as well as in the VR condition. Most importantly, a RS effect specific to VR was obtained at frontal and midfrontal electrode sites that did not occur in the PC condition for a late time window. Electrotomographic results (VARETA) revealed the involvement of a broad cortical network during this time window in VR, suggesting more extensive and sophisticated perceptual and encoding processes under realistic virtual conditions compared to conventional laboratory conditions.

### 4.1. General differences in ERPs between the VR and the PC conditions

The overall lower ERP deflections in the VR condition in comparison to the PC condition further support the assumption of a more natural distribution of cognitive resources under realistic virtual conditions, i.e., cognitive processing characteristics corresponding to those applied in real environments (Schöne et al., 2021b). Remarkably, the larger P1 deflections in the PC condition are counterintuitive, as normally smaller stimuli elicit smaller P1 deflections compared to larger stimuli (Busch et al., 2004; Bayer et al., 2012). While the VR objects remained in their actual dimensions, the 2D objects were scaled to a smaller standardized size as common in conventional laboratory research. Accordingly, if only these minor perceptual differences, i.e., the described size-related differences, would manifest in the results, smaller P1 deflections would have been expected in the PC condition compared to the VR condition. Interestingly, our ERP results point in the contrary direction and therefore suggest that investigating cognitive mechanisms in virtual environments provides further insight into real-life cognition that might go beyond basic visual processing.

Larger ERP deflections in the P1 and N1 components index attentional gain control mechanisms for attended stimuli (Hillyard et al., 1998; Luck et al., 2000). Due to their simplified physical properties, two-dimensional stimuli lack depth information, leading to a higher cognitive load for object recognition (Dan and Reiner, 2017) and to the allocation of more cognitive resources, indexed by the larger ERP deflections in the PC condition. In contrast, processing these features in VR is more effortless due to the three-dimensional virtual environment and therefore requires fewer attentional resources (Dan and Reiner, 2017; Schöne et al., 2021b). In line with these findings, the lower ERP deflections in the VR condition suggest an optimized distribution of cognitive resources in realistic virtual environments on a neuronal level.

### 4.2. Effects of perceptual differences between low-level features of the VR and PC conditions on the RS effect

In contrast to the PC condition, environmental features of the stimuli were maintained and the objects were presented in their real dimensions in the VR condition. The potential influence of these and further low-level perceptual differences such as luminance and color on object perception was accounted by using the respective stimuli in each condition as a perceptual baseline. To this end we calculated the RS effect within the conditions, and subsequently compared these RS effects between conditions. Therefore, differences in the RS effect between the conditions cannot be attributed to distinct low-level perceptual processing, but to distinct mnemonic processing.

### 4.3. RS effect in electrode space

The typical RS effect at the L1 complex could be replicated in both the PC and the VR conditions, indicating the feasibility of the experimental design under both conditions. The sharpening of cortical stimulus representation and the associated decrease in neuronal response to a second presentation (e.g., Desimone, 1996; Gruber et al., 2004) occur under both conditions. Therefore, the cognitive sharpening mechanisms reflected in the typical RS effect can be transferred to more complex realistic environments, generally highlighting the relevance of conventional laboratory research findings.

However, our main finding, the specific RS effect in the VR condition at the late L2 complex, implies more complex but more sophisticated functional properties of perceptual and mnemonic encoding mechanisms for realistic virtual stimuli that go beyond what is captured by the conventional laboratory. The profound engrams formed under realistic virtual conditions, indicated by the specific RS effect in the VR condition, facilitate the integration of experiences into intertwined AB traces.

Interestingly, the first object presentation in the VR condition elicited a more negative ERP amplitude compared to the second presentation in VR and both presentations in the PC condition during L2 at frontal electrodes. Larger ERP deflections are associated with deeper encoding at latencies beyond 200 ms (Guo et al., 2004). Visual information of complex three-dimensional stimuli need to be combined to form a coherent picture (Welchman et al., 2005), leading to more elaborate encoding processes. Given the multimodal nature of virtual stimuli, i.e., visual complexity due to three-dimensionality and sensorimotor aspects related to action execution processing, these processes require a more entangled multisensory integration, which is reflected by larger ERP deflections during encoding of multisensory compared to unisensory stimuli (Xie et al., 2017). Effective multisensory integration depends on higher cognitive processes (Freiherr et al., 2013) involving the coordination of sensorimotor, motivational, and self-referential mechanisms. The realistic virtual stimuli leave the impression to be graspable and interactive, and the mere intention to move modulates early visual cortical activity (Gallivan et al., 2019). Therefore, automatic motor preparation in response to real objects (Marini et al., 2019) might be one factor enhancing encoding mechanisms in realistic virtual scenarios.

Furthermore, the general possibility to interact with the virtual surrounding and the sensorimotor activation are accompanied by motivational responses in terms of approach motivation toward the realistic objects, which is not promoted by the simple stimuli in the PC condition. Realistic virtual in comparison to conventional 2D stimuli elicited partially contrasting motivational-related cortical activity patterns (Schöne et al., 2021a). These findings suggest distinct motivational processes influencing cortical activity during perceptual processing even for neutral scenes.

RS as a correlate of priming and encoding processes provides information about the first instance of forming a memory trace. Therefore, the specific RS effect indicates that the formation of self-relevant engrams from VR experiences that are functionally associated with AB (Schöne et al., 2019) might be facilitated by the extensive encoding mechanism in VR, which includes the multisensory integration of multimodal virtual stimuli. The egocentric perspective, the physical vicinity, and the perceived interactivity of realistic virtual stimuli might increase the personal relevance of an object. The latter is already assessed during object perception (Barrett and Bar, 2009). A self-related focus during encoding influences ERP deflections at later time windows (Leynes and Crawford, 2018), providing further evidence for a potential impact of self-relevance on encoding mechanisms reflected in the late L2 complex in realistic virtual environments. Concludingly, the previously observed relation of memory for VR experiences to AB occurs at early encoding stages.

### 4.4. RS effect in source space

The source analysis revealed multiple cortical generators for the genuine RS effect in VR during L2 corresponding to the hypothesized complex integration process during encoding in realistic virtual environments as indicated by the ERPs. These findings further suggest the involvement of a broader network of cortical areas during the encoding of virtual as opposed to conventional laboratory objects. The sources of the genuine RS effects in the PC and VR conditions indicate the cortical generators which are specific for the respective condition.

Distinct parts of the right inferior temporal gyrus were involved in generating the genuine RS effects during object perception. The inferior temporal gyrus was previously observed as a generator of the typical RS effect (Gruber et al., 2006a) and is part of the ventral visual pathway, responsible for object perception (for review see Kravitz et al., 2013). Therefore, the genuine RS effect in the PC condition is generated by typical areas involved in visual object processing (Desimone et al., 1984; Aggelopoulos et al., 2005; Conway, 2018) as well as in mnemonic processing of objects (Mishkin, 1982; Miyashita, 1993; Owen et al., 1996). In comparison to the part of the inferior temporal gyrus generating the genuine RS effect in the VR condition, the generator for the genuine RS effect in the PC condition is located more anteriorly. Within the ventral visual pathway, information is projected from the striate cortex to prestriate regions and from there to the inferior temporal cortex (Ungerleider and Mishkin, 1982). Therefore, during L2, the processing of objects in the PC condition is farther proceeded in the ventral pathway as opposed to the processing in the VR condition.

The right middle temporal gyrus, the left inferior temporal gyrus, the left lateral occipitotemporal gyrus and the lingual gyrus were identified as additional cortical generators for the genuine RS effect in the VR condition. This broadly distributed activity indicates a multimodal integration process during the encoding of realistic objects.

Beyond general object processing in the ventral visual stream indicated in both conditions by the activity of the right inferior temporal gyrus, the left inferior temporal gyrus was observed to be relevant for semantic knowledge (Chan et al., 2001; Giovanello et al., 2003; Antonucci et al., 2008). Thus, in realistic environments, not only the visual aspects are processed more extensively, but also the semantic information of the objects. Among other information, the visual system processes semantic aspects by evaluating the object’s relation to the scene, i.e., whether the object is consistent with the scene (Wu et al., 2014). Since the setup in the PC condition is simplified, the objects do not have to be compared to the rest of the scene. On the contrary, the virtual objects placed on the table can be brought in line with their surrounding which requires additional cognitive processes. Therefore, the activation of the left inferior temporal gyrus suggests the engagement of additional higher cognitive processes for object perception in complex realistic environments.

The three-dimensionality and the presentation within an arm’s length makes the VR objects appear realistic and graspable. Interestingly, this possible interactivity is reflected in the activity of the left lateral occipitotemporal gyrus (or fusiform gyrus) which connects regions involved in action-related processing (Bracci et al., 2012). In particular, this cortical region is engaged in encoding grasping-related properties (Wu et al., 2020), is associated with visual hand representation (Bracci et al., 2010) and reacts to objects that can actively be used (Bracci and Peelen, 2013). Most importantly, the left lateral occipitotemporal gyrus is not only associated with the processing of tangible objects but is relevant for the integration of multisensory information of those objects (Kassuba et al., 2011).

The left lingual gyrus and the middle temporal gyrus, which are also involved in generating the genuine RS effect in VR, are engaged in the mnemonic processing of realistic objects. While activity of the left lingual gyrus is associated with visual memory (Kozlovskiy et al., 2014), the right middle temporal gyrus is relevant for AB, in particular it contributes to the retrieval of the situation’s encoding context (Noulhiane et al., 2007). As typical for the conventional laboratory, the 2D objects in the PC condition were stripped of their context which was replaced by a gray background, whereas the 3D objects in VR were presented within a coherent contextual setting. In conclusion, the activity of the right middle temporal gyrus during encoding of more complex VR environments suggests the involvement of cognitive processes associated with AB during early stages of memory trace formation.

## 5. Conclusion

In conclusion, the results regarding both the specific RS effect indicated by ERPs and the genuine RS effect indicated by the source analysis in VR reveal the activation of a broad cortical network during the encoding of realistic objects. This network is engaged in higher cognitive processing, the integration of multimodal sensory information and the formation of memory traces. Although multisensory integration requires complex higher cognitive processing, the broad cortical network benefits from subsequent sharpening mechanisms associated with RS, facilitating encoding processes in realistic environments. The results suggest that the perception of an object under realistic virtual conditions is not limited to an isolated visual process, but rather consists of an interconnected functional system responsible for multisensory integration. As opposed to the laboratory, the formation of engrams for objects under realistic conditions involves more extensive encoding mechanisms indexed by the specific RS effect in VR, which might facilitate the formation of more intertwined AB memory traces. Moreover, our ERP results further support the assumption that cognitive resources are more naturally and optimally distributed under realistic conditions. While previous conventional laboratory research mainly focused on isolated cognitive processes, future research should consider to investigate them in a more comprehensive perspective. It is essential to extend laboratory findings under more realistic conditions to fully understand the functional properties of natural cognitive mechanisms within their entire network.

## 6. Limitations and future directions

It should be considered that our source analysis only provides statistically approximate results concerning the involved brain areas. Previous fMRI research regarding the typical RS effect revealed that several subcortical brain regions show RS as well (Habeck et al., 2006), which cannot be captured by EEG measurements. Therefore, an approach combining our EEG findings with fMRI research could provide further insight into mnemonic processing in realistic virtual environments.

Additionally, in several typical studies, the RS effect has been observed in the gamma frequency band, which correlates with indirect memory (e.g., Gruber and Müller, 2002, 2006; Gruber et al., 2004). While our study focused on the ERP results, the investigation of the RS effect in the gamma frequency band in VR could be the following step to further understand cortical object representation under realistic conditions.

## Data availability statement

The datasets presented in this study can be found in online repositories. The names of the repository/repositories and accession number(s) can be found at: https://osf.io/bfcu9/?view_only=0dd687e8cbf348ffbea010ddfeb33d8a.

## Ethics statement

The studies involving human participants were reviewed and approved by the local ethic committee of Osnabrueck University, Germany. The patients/participants provided their written informed consent to participate in this study.

## Author contributions

BS developed the 2D version of the experiment, MJ developed the Unity VR environment, and JK integrated the EEG applications. Testing and data collection were performed by BS, JK, and MJ. ERP data analyses were performed by BS and source analyses were performed by JK under the supervision of BS and TG. BS and MJ drafted the manuscript and JK provided critical revisions. All authors approved the final version of the manuscript for submission.

## Funding

This research was funded by the MWK Niedersachsen and the VolkswagenStiftung grant number 11-76251-14-1/21. We acknowledge support by Deutsche Forschungsgemeinschaft (DFG) and Open Access Publishing Fund of Osnabrück University.

## Conflict of interest

The authors declare that the research was conducted in the absence of any commercial or financial relationships that could be construed as a potential conflict of interest.

## Publisher’s note

All claims expressed in this article are solely those of the authors and do not necessarily represent those of their affiliated organizations, or those of the publisher, the editors and the reviewers. Any product that may be evaluated in this article, or claim that may be made by its manufacturer, is not guaranteed or endorsed by the publisher.

## Footnotes

^1^ https://www.vive.com/de/product/vive-cosmos/overview/

^2^ https://github.com/sccn/labstreaminglayer

## References

[ref1] Aggelopoulos N. C. Franco L. Rolls E. T. (2005). Object perception in natural scenes: encoding by inferior temporal cortex simultaneously recorded neurons. J. Neurophysiol. 93, 1342–1357. doi: 10.1152/jn.00553.2004, PMID: 15496489

[ref2] Antonucci S. M. Beeson P. M. Labiner D. M. Rapcsak S. Z. (2008). Lexical retrieval and semantic knowledge in patients with left inferior temporal lobe lesions. Aphasiology 22, 281–304. doi: 10.1080/02687030701294491, PMID: 19756227 PMC2743433

[ref3] Barrett L. F. Bar M. (2009). See it with feeling: affective predictions during object perception. Philos. Trans. R Soc. Lond. B: Biol. Sci. 364, 1325–1334. doi: 10.1098/rstb.2008.0312, PMID: 19528014 PMC2666711

[ref4] Bayer M. Sommer W. Schacht A. (2012). Font size matters-emotion and attention in cortical responses to written words. PLoS One 7, 1–6. doi: 10.1371/journal.pone.0036042, PMID: 22590518 PMC3348912

[ref5] Bosch-Bayard D. J. Valdés-Sosa P. Virues-Alba T. Aubert-Vázquez E. John D. E. R. Harmony T.. (2001). 3D statistical parametric mapping of EEG source spectra by means of variable resolution electromagnetic tomography (VARETA). Clin. Electroencephalogr. 32, 47–61. doi: 10.1177/155005940103200203, PMID: 11360721

[ref6] Bracci S. Cavina-Pratesi C. Ietswaart M. Caramazza A. Peelen M. V. (2012). Closely overlapping responses to tools and hands in left lateral occipitotemporal cortex. J. Neurophysiol. 107, 1443–1456. doi: 10.1152/jn.00619.2011, PMID: 22131379

[ref7] Bracci S. Ietswaart M. Peelen M. V. Cavina-Pratesi C. (2010). Dissociable neural responses to hands and non-hand body parts in human left extrastriate visual cortex. J. Neurophysiol. 103, 3389–3397. doi: 10.1152/jn.00215.2010, PMID: 20393066 PMC2888254

[ref8] Bracci S. Peelen M. V. (2013). Body and object effectors: the organization of object representations in high-level visual cortex reflects body-object interactions. J. Neurosci. 33, 18247–18258. doi: 10.1523/JNEUROSCI.1322-13.2013, PMID: 24227734 PMC6619748

[ref9] Busch N. A. Debener S. Kranczioch C. Engel A. K. Herrmann C. S. (2004). Size matters: effects of stimulus size, duration and eccentricity on the visual gamma-band response. Clin. Neurophysiol. 115, 1810–1820. doi: 10.1016/j.clinph.2004.03.015, PMID: 15261860

[ref10] Cabeza R. Prince S. E. Daselaar S. M. Greenberg D. L. Budde M. Dolcos F.. (2004). Brain activity during episodic retrieval of autobiographical and laboratory events: an fMRI study using a novel photo paradigm. J. Cogn. Neurosci. 16, 1583–1594. doi: 10.1162/0898929042568578, PMID: 15622612

[ref11] Cabeza R. St Jacques P. (2007). Functional neuroimaging of autobiographical memory. Trends Cogn. Sci. 11, 219–227. doi: 10.1016/j.tics.2007.02.005 17382578

[ref12] Chan D. Fox N. C. Scahill R. I. Crum W. R. Whitwell J. L. Leschziner G.. (2001). Patterns of temporal lobe atrophy in semantic dementia and Alzheimer’s disease. Ann. Neurol. 49, 433–442. doi: 10.1002/ana.92 11310620

[ref13] Chirico A. Ferrise F. Cordella L. Gaggioli A. (2018). Designing awe in virtual reality: an experimental study. Front. Psychol. 8:2351. doi: 10.3389/fpsyg.2017.02351, PMID: 29403409 PMC5786556

[ref14] Conway M. A. (2005). Memory and the self. J. Mem. Lang. 53, 594–628. doi: 10.1016/j.jml.2005.08.005

[ref15] Conway B. R. (2018). The organization and operation of inferior temporal cortex. Annu. Rev. Vis. Sci. 4, 381–402. doi: 10.1146/annurev-vision-091517-034202, PMID: 30059648 PMC6404234

[ref16] Dan A. Reiner M. (2017). EEG-based cognitive load of processing events in 3D virtual worlds is lower than processing events in 2D displays. Int. J. Psychophysiol. 122, 75–84. doi: 10.1016/j.ijpsycho.2016.08.013, PMID: 27592084

[ref17] Delorme A. Makeig S. (2004). EEGLAB: an open source toolbox for analysis of single-trial EEG dynamics including independent component analysis. J. Neurosci. Methods 134, 9–21. doi: 10.1016/j.jneumeth.2003.10.009, PMID: 15102499

[ref18] Desimone R. (1996). Neural mechanisms for visual memory and their role in attention. Proc. Natl. Acad. Sci. U. S. A. 93, 13494–13499. doi: 10.1073/pnas.93.24.13494, PMID: 8942962 PMC33636

[ref19] Desimone R. Albright T. D. Gross C. G. Bruce C. (1984). Stimulus-selective properties of inferior temporal neurons in the macaque. J. Neurosci. 4, 2051–2062. doi: 10.1523/JNEUROSCI.04-08-02051.1984, PMID: 6470767 PMC6564959

[ref20] Di Russo F. Martínez A. Sereno M. I. Pitzalis S. Hillyard S. A. (2002). Cortical sources of the early components of the visual evoked potential. Hum. Brain Mapp. 15, 95–111. doi: 10.1002/hbm.10010, PMID: 11835601 PMC6871868

[ref21] Evans A. C. Collins D. L. Mills S. R. Brown E. D. Kelly R. L. Peters T. M. (1993). “3D statistical neuroanatomical models from 305 MRI volumes.” in *1993 IEEE Conference Record Nuclear Sci Symp and Med Imaging Conference*, 1813–1817.

[ref22] Freiherr J. Lundström J. N. Habel U. Reetz K. (2013). Multisensory integration mechanisms during aging. Front. Hum. Neurosci. 7:863. doi: 10.3389/fnhum.2013.00863, PMID: 24379773 PMC3861780

[ref23] Gallivan J. P. Chapman C. S. Gale D. J. Flanagan J. R. Culham J. C. (2019). Selective modulation of early visual cortical activity by movement intention. Cereb. Cortex 29, 4662–4678. doi: 10.1093/cercor/bhy345, PMID: 30668674 PMC6917518

[ref24] Giovanello K. S. Alexander M. Verfaellie M. (2003). Differential impairment of person-specific knowledge in a patient with semantic dementia. Neurocase 9, 15–26. doi: 10.1076/neur.9.1.15.14369, PMID: 16210222

[ref25] Gruber T. Giabbiconi C. M. Trujillo-Barreto N. J. Müller M. M. (2006a). Repetition suppression of induced gamma band responses is eliminated by task switching. Eur. J. Neurosci. 24, 2654–2660. doi: 10.1111/j.1460-9568.2006.05130.x, PMID: 17100853

[ref26] Gruber T. Malinowski P. Müller M. M. (2004). Modulation of oscillatory brain activity and evoked potentials in a repetition priming task in the human EEG. Eur. J. Neurosci. 19, 1073–1082. doi: 10.1111/j.0953-816X.2004.03176.x, PMID: 15009155

[ref27] Gruber T. Müller M. M. (2002). Effects of picture repetition on induced gamma band responses, evoked potentials, and phase synchrony in the human EEG. Cogn. Brain Res. 13, 377–392. doi: 10.1016/S0926-6410(01)00130-6, PMID: 11919002

[ref28] Gruber T. Müller M. M. (2006). Oscillatory brain activity in the human EEG during indirect and direct memory tasks. Brain Res. 1097, 194–204. doi: 10.1016/j.brainres.2006.04.069, PMID: 16729980

[ref29] Gruber T. Trujillo-Barreto N. J. Giabbiconi C.-M. Valdés-Sosa P. A. Müller M. M. (2006b). Brain electrical tomography (BET) analysis of induced gamma band responses during a simple object recognition task. NeuroImage 29, 888–900. doi: 10.1016/j.neuroimage.2005.09.004, PMID: 16242965

[ref30] Guo C. Zhu Y. Ding J. Fan S. Paller K. A. (2004). An electrophysiological investigation of memory encoding, depth of processing, and word frequency in humans. Neurosci. Lett. 356, 79–82. doi: 10.1016/j.neulet.2003.09.049, PMID: 14746868

[ref31] Habeck C. Hilton H. J. Zarahn E. Brown T. Stern Y. (2006). An event-related fMRI study of the neural networks underlying repetition suppression and reaction time priming in implicit visual memory. Brain Res. 1075, 133–141. doi: 10.1016/j.brainres.2005.11.102, PMID: 16476414

[ref32] Hillyard S. A. Vogel E. K. Luck S. J. (1998). Sensory gain control (amplification) as a mechanism of selective attention: electrophysiological and neuroimaging evidence. Philos. Trans. R Soc. Lond. B: Biol. Sci. 353, 1257–1270. doi: 10.1098/rstb.1998.0281, PMID: 9770220 PMC1692341

[ref33] Holler D. E. Fabbri S. Snow J. C. (2020). Object responses are highly malleable, rather than invariant, with changes in object appearance. Sci. Rep. 10, 4654–4614. doi: 10.1038/s41598-020-61447-8, PMID: 32170123 PMC7070005

[ref34] Kassuba T. Klinge C. Hölig C. Menz M. M. Ptito M. Röder B.. (2011). The left fusiform gyrus hosts trisensory representations of manipulable objects. NeuroImage 56, 1566–1577. doi: 10.1016/j.neuroimage.2011.02.032, PMID: 21334444

[ref35] Kisker J. Gruber T. Schöne B. (2021a). Experiences in virtual reality entail different processes of retrieval as opposed to conventional laboratory settings: a study on human memory. Curr. Psychol. 40, 3190–3197. doi: 10.1007/s12144-019-00257-2

[ref36] Kisker J. Gruber T. Schöne B. (2021b). Virtual reality experiences promote autobiographical retrieval mechanisms: electrophysiological correlates of laboratory and virtual experiences. Psychol. Res. 85, 2485–2501. doi: 10.1007/s00426-020-01417-x, PMID: 32930880 PMC8440245

[ref37] Kozlovskiy S. A. Pyasik M. M. Korotkova A. V. Vartanov A. V. Glozman J. M. Kiselnikov A. A. (2014). Activation of left lingual gyrus related to working memory for schematic faces. Int. J. Psychophysiol. 94:241. doi: 10.1016/j.ijpsycho.2014.08.928

[ref38] Kravitz D. J. Saleem K. S. Baker C. I. Ungerleider L. G. Mishkin M. (2013). The ventral visual pathway: an expanded neural framework for the processing of object quality. Trends Cogn. Sci. 17, 26–49. doi: 10.1016/j.tics.2012.10.011, PMID: 23265839 PMC3532569

[ref39] Leynes P. A. Crawford C. J. (2018). Event-related potential (ERP) evidence that encoding focus alters recollected features. Brain Cogn. 127, 42–50. doi: 10.1016/j.bandc.2018.09.005, PMID: 30253265

[ref40] Luck S. J. Woodman G. F. Vogel E. K. (2000). Event-related potential studies of attention. Trends Cogn. Sci. 4, 432–440. doi: 10.1016/S1364-6613(00)01545-X 11058821

[ref41] Marini F. Breeding K. A. Snow J. C. (2019). Distinct visuo-motor brain dynamics for real-world objects versus planar images. NeuroImage 195, 232–242. doi: 10.1016/j.neuroimage.2019.02.026, PMID: 30776529 PMC6536332

[ref42] Martens U. Trujillo-Barreto N. Gruber T. (2011). Perceiving the tree in the woods: segregating brain responses to stimuli constituting natural scenes. J. Neurosci. 31, 17713–17718. doi: 10.1523/JNEUROSCI.4743-11.2011, PMID: 22131431 PMC6623807

[ref43] Mishkin M. (1982). A memory system in the monkey. Philos. Trans. R Soc. Lond. B Biol. Sci. 298, 85–95. doi: 10.1098/rstb.1982.0074 6125978

[ref44] Miyashita Y. (1993). Inferior temporal cortex: where visual perception meets memory. Annu. Rev. Neurosci. 16, 245–263. doi: 10.1146/annurev.ne.16.030193.001333, PMID: 8460893

[ref45] Noulhiane M. Piolino P. Hasboun D. Clemenceau S. Baulac M. Samson S. (2007). Autobiographical memory after temporal lobe resection: neuropsychological and MRI volumetric findings. Brain 130, 3184–3199. doi: 10.1093/brain/awm258, PMID: 17986479

[ref46] Ogmen H. Shibata K. Yazdanbakhsh A. (2020). Perception, cognition, and action in hyperspaces: implications on brain plasticity, learning, and cognition. Front. Psychol. 10:3000. doi: 10.3389/fpsyg.2019.03000, PMID: 32038384 PMC6987450

[ref47] Owen A. M. Milner B. Petrides M. Evans A. C. (1996). Memory for object features versus memory for object location: a positron-emission tomography study of encoding and retrieval processes. Proc. Natl. Acad. Sci. U. S. A. 93, 9212–9217. doi: 10.1073/pnas.93.17.9212, PMID: 8799180 PMC38621

[ref48] Parsons T. D. (2015). Virtual reality for enhanced ecological validity and experimental control in the clinical, affective and social neurosciences. Front. Hum. Neurosci. 9:600. doi: 10.3389/fnhum.2015.00660, PMID: 26696869 PMC4675850

[ref49] Rugg M. D. Soardi M. Doyle M. C. (1995). Modulation of event-related potentials by the repetition of drawings of novel objects. Cogn. Brain Res. 3, 17–24. doi: 10.1016/0926-6410(95)00014-3, PMID: 8719018

[ref50] Schöne B. Kisker J. Sylvester R. S. Radtke E. L. Gruber T. (2021a). Library for universal virtual reality experiments (luVRe): a standardized immersive 3D/360° picture and video database for VR based research. Curr. Psychol. doi: 10.1007/s12144-021-01841-1

[ref51] Schöne B. Köster M. Gruber T. (2018). Coherence in general and personal semantic knowledge: functional differences of the posterior and centro-parietal N400 ERP component. Exp. Brain Res. 236, 2649–2660. doi: 10.1007/s00221-018-5324-1, PMID: 29974147

[ref52] Schöne B. Schomberg J. Gruber T. Quirin M. (2015). Event-related frontal alpha asymmetries: electrophysiological correlates of approach motivation. Exp. Brain Res. 234, 559–567. doi: 10.1007/s00221-015-4483-6, PMID: 26537961

[ref53] Schöne B. Sylvester R. S. Radtke E. L. Gruber T. (2021b). Sustained inattentional blindness in virtual reality and under conventional laboratory conditions. Virtual Reality 25, 209–216. doi: 10.1007/s10055-020-00450-w

[ref54] Schöne B. Wessels M. Gruber T. (2019). Experiences in virtual reality: a window to autobiographical memory. Curr. Psychol. 38, 715–719. doi: 10.1007/s12144-017-9648-y

[ref56] Shamay-Tsoory S. G. Mendelsohn A. (2019). Real-life neuroscience: an ecological approach to brain and behavior research. Perspect. Psychol. Sci. 14, 841–859. doi: 10.1177/1745691619856350, PMID: 31408614

[ref57] Smith S. A. (2019). Virtual reality in episodic memory research: a review. Psychon. Bull. Rev. 26, 1213–1237. doi: 10.3758/s13423-019-01605-w, PMID: 31037605

[ref58] Snow J. C. Culham J. C. (2021). The treachery of images: how realism influences brain and behavior. Trends Cogn. Sci. 25, 506–519. doi: 10.1016/j.tics.2021.02.008, PMID: 33775583 PMC10149139

[ref59] Sommer V. R. Mount L. Weigelt S. Werkle-Bergner M. Sander M. C. (2021). Memory specificity is linked to repetition effects in event-related potentials across the lifespan. Dev. Cogn. Neurosci. 48:100926. doi: 10.1016/j.dcn.2021.100926, PMID: 33556880 PMC7868631

[ref60] Stefanics G. Heinzle J. Czigler I. Valentini E. Stephan K. E. (2020). Timing of repetition suppression of event-related potentials to unattended objects. Eur. J. Neurosci. 52, 4432–4441. doi: 10.1111/ejn.13972, PMID: 29802671 PMC7818225

[ref61] Trujillo-Barreto N. J. Aubert-Vázquez E. Valdés-Sosa P. A. (2004). Bayesian model averaging in EEG/MEG imaging. NeuroImage 21, 1300–1319. doi: 10.1016/j.neuroimage.2003.11.008, PMID: 15050557

[ref62] Ungerleider L. G. Mishkin M. (1982). “Two cortical visual systems,” Analysis of Visual Behavior. eds. Goodale M. A. Ingle D. J. Mansfield R. J. W. (Cambridge: MIT Press), 549–586.

[ref63] Wedel M. Pieters R. (2007). A review of eye-tracking research in marketing. Rev. Market. Res. 4, 123–147. doi: 10.1108/S1548-6435(2008)0000004009

[ref64] Welchman A. E. Deubelius A. Conrad V. Bülthoff H. H. Kourtzi Z. (2005). 3D shape perception from combined depth cues in human visual cortex. Nat. Neurosci. 8, 820–827. doi: 10.1038/nn1461, PMID: 15864303

[ref65] Wiggs C. L. Martin A. (1998). Properties and mechanisms of perceptual priming. Curr. Opin. Neurobiol. 8, 227–233. doi: 10.1016/S0959-4388(98)80144-X 9635206

[ref66] Worsley K. J. Liao C. H. Aston J. Petre V. Duncan G. H. Morales F.. (2002). A general statistical analysis for fMRI data. NeuroImage 15, 1–15. doi: 10.1006/nimg.2001.0933, PMID: 11771969

[ref67] Worsley K. J. Marrett S. Neelin P. Vandal A. C. Friston K. J. Evans A. C. (1996). A unified statistical approach for determining significant signals in images of cerebral activation. Hum. Brain Mapp. 4, 58–73. doi: 10.1002/(SICI)1097-0193(1996)4:1<58::AID-HBM4>3.0.CO;2-O, PMID: 20408186

[ref68] Wu W. Wang X. Wei T. He C. Bi Y. (2020). Object parsing in the left lateral occipitotemporal cortex: whole shape, part shape, and graspability. Neuropsychologia 138:107340:107340. doi: 10.1016/j.neuropsychologia.2020.107340, PMID: 31935393

[ref69] Wu C.-C. Wick F. A. Pomplun M. (2014). Guidance of visual attention by semantic information in real-world scenes. Front. Psychol. 5:54. doi: 10.3389/fpsyg.2014.00054, PMID: 24567724 PMC3915098

[ref70] Xie Y. Xu Y. Bian C. Li M. (2017). Semantic congruent audiovisual integration during the encoding stage of working memory: an ERP and sLORETA study. Sci. Rep. 7, 1–10. doi: 10.1038/s41598-017-05471-1, PMID: 28698594 PMC5505990

