# Supplementary material for: Comparing encoding mechanisms in realistic virtual reality and conventional 2D laboratory settings: Event-related potentials in a repetition suppression paradigm
Source: Front Psychol. 2023 Jan 27;14:1051938. doi: 10.3389/fpsyg.2023.1051938 (PMC9912617; doi:10.3389/fpsyg.2023.1051938)
Supplement: Supplementary file 1 [file Data_Sheet_1.PDF]

Supplementary Material

S1

List of objects used as stimuli.

1. Thermos mug black
2. Tea tin
3. Salt/pepper mill
4. Duct tape
5. Tool box blue
6. Scissors
7. Headphones blue/grey
8. Coffeepot
9. TV remote
10. Thestral figurine
11. Power strip
12. Stuffed spaghetti monster
13. Stuffed squid
14. Sunglasses
15. Flowers in vase
16. Lunch box green
17. Ice cube form
18. Mini camera
19. Mug pink
20. Tee strainer
21. Bowl purple/white
22. Bedroom slipper
23. Pencil case pink
24. Grater
25. Yoga block
26. Smoothie bottle
27. Folding rule
28. Milk frother
29. Ship figurine
30. Thermos mug colorful
31. Ballet figurine 1
32. Plastic plant 1
33. Bamboo plant pot
34. Hair brush
35. Plastic plant blue
36. Hair detangler
37. Tee package
38. Cotton swab
39. Hair clip
40. Tealight holder glass
41. Candle red
42. Stuffed flamingo
43. Smartphone stand silver
44. Hawaiian lei necklace
45. Plastic rose 1
46. Pencil case colorful
47. Pointe shoe
48. Ballet slipper black
49. Wood log
50. Dance sneaker
51. Jewelry box
52. Ballet figurine 2
53. Banana
54. Washcloth
55. Banana lunch box
56. Sandal beige
57. Lunch box pink
58. Camera battery
59. Jar of oats
60. Headband
61. Gloves black
62. Striped clay jug
63. Glasses case black
64. Lunch box yellow
65. Plastic rose 2
66. Fine strainer
67. Wool socks
68. Top
69. Small drinking bottle metallic
70. Vegetable stock
71. Loaf pan
72. Cloth striped
73. Towel
74. Tissue box
75. Trophy
76. Electric bell
77. Shoe brush
78. Workout item
79. Toy horse
80. Stuffed bear blue
81. Plastic cup green
82. Dish soap
83. Cloth green
84. Dish towel green/white

85. Coffee tin metal
86. Mug orange
87. Knife
88. Spoon
89. Fork
90. Blackboard sponge
91. Large decoration heart
92. Dustpan blue
93. Hand brush
94. Lint roller
95. Gloves grey
96. Umbrella
97. Purse
98. Picture frame white
99. Picture frame brown
100. Beanie
101. Duster
102. Lid
103. Carton box
104. Sponge
105. Tambourine
106. Dish towel blue/white
107. Dishcloth blue dotted
108. Pasta strainer
109. Mixing bowl
110. Shoes grey
111. Piece of wood
112. Plastic flowers purple
113. Respiratory mask
114. Wooden plant pot
115. Metallic plant pot
116. Tissue box empty
117. Plastic plant pot
118. Drinking bottle grey
119. Wallet
120. Plastic pipe
121. Rolling pin
122. Salt mill
123. Plastic plate orange
124. Potato masher
125. Hammer
126. Food processor
127. Hand brush white
128. Dustpan red
129. Baking tin round metal
130. Baking tin round silicon
131. Music box
132. Green jewelry box
133. Lemon squeezer blue
134. Punch
135. Shell figurine
136. Booklet green
137. Blue box
138. Apple
139. Pear
140. Salat lunch box
141. Small toilet bag
142. Fitness band
143. Wooden turtle
144. Rooster figurine
145. Painting brush
146. Hair dryer
147. Cable bag
148. Small bin
149. Sauce boat
150. Bottle brush
151. Dish brush
152. Hanger white
153. Tablet case
154. Tablet
155. Toilet paper
156. Mug with lid
157. Make-up brush
158. Wooden horse with sleigh
159. Soap dish
160. Bicycle bag
161. Lemon squeezer beige
162. Carton box colorful
163. Dog bowl
164. Heated slippers
165. Oven dish
166. Bicycle lock
167. Purse pink
168. Drinking bottle yellow
169. Perfume
170. Wooden spoon
171. Spatula black
172. Spatula white
173. Small painting brush
174. Dough scraper pink
175. Knife black
176. Boulder shoe
177. Muffin tin
178. Lunchbox turquoise
179. Lighthouse light chain
180. Plant pot purple

181. Plant pot pink
182. Small plant
183. Wooden dish brush
184. Porcelain plate flowers
185. Kitchen paper roll
186. Bowl blue
187. Bowl beige
188. Aluminium foil
189. Cloth blue
190. Candle white
191. Baby powder
192. Light chain
193. Polaroid camera
194. Basket
195. Champagne
196. Coffee machine
197. Controller
198. Headphones blue/ black
199. Laptop carton
200. Coffee pot black
201. Graduation hat
202. Starwars figurine
203. Big punch black
204. Cable white
205. Blue folder
206. Bedside lamp
207. Fabric bag
208. Make-up bag black
209. Paintbox
210. Ribbon roll
211. Christmas decoration
212. Star candle holder
213. Shower wiper
214. Plant pot white
215. Bottle cover
216. Sellotape roll
217. Fan
218. Buddha figurine
219. Laptop
220. Measuring instrument
221. Alarm clock
222. Stapler black
223. Pencil box
224. Can opener
225. Toolbox green
226. Measuring tape
227. Stuffed Pokémon ball
228. Small shovel
229. Small rake
230. Wool ball
231. Insect house
232. Napkin holder
233. Pencil case blue
234. Calculator case
235. Calculator
236. Small vase blue
237. Small box purple
238. Rattan ball light chain
239. Pencil case red
240. Ribbon package
241. Wooden star
242. Weight cuff
243. Dumbbell
244. Fascia Roller
245. Glass with straw
246. Cable green
247. Big candle purple
248. Hot glue gun
249. Dustpan metal
250. Charging cable black
251. Hard disk
252. Saw
253. Wood glue
254. Computer mouse
255. Pliers red
256. Book black
257. First aid bag
258. Paint roller
259. Plastic mug red
260. Pepper mill
261. Hanger blue
262. Round box blue
263. Toaster attachment
264. Spice jar
265. House figurine white
266. Gift bag
267. Notebook
268. Cash box
269. Autumn decoration
270. Radio clock
271. Coffee tin brown/ white
272. Red candle in glass
273. Pot
274. Whisk
275. Pizza board
276. House figurine blue
277. Lighter
278. Plant 1

279. Plant 2
280. Plant 3
281. Plant 4
282. Plant 5
283. Plant 6
284. Plant 7
285. Egg carton
286. Orange
287. Board game
288. Scarf
289. Foot massage roller
290. Chalk
291. Bicycle helmet
292. Soap dispenser
293. Steam iron
294. Carton box white/ green
295. Paper bag
296. Small decoration cabinet
297. Birdhouse
298. Clay vase
299. Plunger
300. Picture frame black
301. Plastic folder
302. Pylon
303. Hot-water bottle
304. Nutcracker
305. Pliers grey
306. Office chair castor
307. Digital clock
308. Toy car
309. Sandal brown
310. Ballet slippers blue/ red
311. Stuffed monkey
312. Box grater
313. Skipping rope
314. Remote control
315. Notepad
316. Plastic plant 2
317. Hand blender attachment
318. Boot brown
319. Fitness band loop
320. Desk calendar
321. Chess board
322. Christmas ornaments
323. Candle purple
324. Hair straightener case
325. Hair straightener
326. Camera bag
327. Camera
328. Metronome
329. Camera flash light
330. Vase white
331. Picture frame green/white
332. Smartphone stand black
333. VHS cassette
334. Make-up bag blue
335. Make-up
336. Seagull figurine
337. Wooden box
338. Manicure set
339. Coffee mill
340. Popsicle mold
341. Pen holder
342. Round notepad
343. Small lantern
344. Stamp pad
345. Watering can
346. Rubber duck
347. Pan
348. Microwave grid
349. Soup ladle
350. Potholder
351. Dough scraper grey
352. Hand mixer attachment
353. Small remote
354. Pizza cutter
355. Bread basket
356. Flip-flop
357. Hanger black
358. Wig stand
359. Hair straightener brush
360. Spider catcher

Table S2

Test statistics for rmANOVA with the factors Modality, Repetition and Cluster for the P1 time window (125-165).

Tests of Within-Subjects Effects[a]

	Type III Sum of Squares	*df*	*Mean Square*	*F*	*p*	η^2
Modality	1.52	1.00	1.52	6.74	.016	0.22
Repetition	<0.01	1.00	<0.01	0.02	.878	<0.01
Cluster	2723.23	1.31	2072.30	50.42	<.001	0.68
Modality*Repetition	0.02	1.00	0.02	0.32	.577	0.01
Modality*Cluster	180.99	1.88	96.06	20.03	<.001	0.45
Repetition*Cluster	0.50	3.66	0.14	0.38	.804	0.02
Modality*Repetition*Cluster	1.34	2.58	0.52	0.72	.525	0.03

a. Greenhouse-Geisser corrected ANOVA

Table S3

Test statistics for post-hoc paired t-tests regarding ERP amplitudes in the PC and the VR condition for each cluster for the P1 time window (125-165ms).

	Paired Samples Statistics		*Paired Samples Test*					
			Paired Differences					
	M	*SD*	*M*	*SD*	*t*	*df*	*p*	*Cohen's d*[a]
PC: frontal	-2.99	1.75	-1.38	1.51	-4.59	24	<.001	-0.918
VR: frontal	-1.60	1.85						
PC: mid-frontal	-2.50	1.59	-1.19	1.05	-5.67	24	<.001	-1.133
VR: mid-frontal	-1.30	1.31						
PC: left temporal	-0.40	0.94	-0.45	0.91	-2.48	24	.021	-0.496
VR: left temporal	0.05	0.93						
PC: right temporal	-0.25	0.98	0.47	0.95	2.48	24	.020	0.497
VR: right temporal	-0.72	0.82						
PC: posterior	5.18	3.14	1.88	1.94	4.86	24	<.001	0.971
VR: posterior	3.30	2.61						
PC: centro-parietal	-0.53	0.77	0.07	0.64	0.58	24	.569	0.116
VR: centro-parietal	-0.61	0.73						

a. Cohen's d uses the sample standard deviation of the mean difference.

Table S4

Test statistics for rmANOVA with the factors Modality, Repetition and Cluster for the N1 time window (165-205ms).

Tests of Within-Subjects Effects[a]

	Type III Sum of Squares	*df*	*Mean Square*	*F*	*p*	η^2
Modality	0.79	1.00	0.79	2.64	.117	0.10
Repetition	0.08	1.00	0.08	1.17	.290	0.05
Cluster	1881.53	1.52	1234.73	37.39	<.001	0.61
Modality*Repetition	0.02	1.00	0.02	0.41	.528	0.02
Modality*Cluster	133.72	2.15	62.23	12.10	<.001	0.34
Repetition*Cluster	4.31	2.57	1.68	1.79	.166	0.07
Modality*Repetition*Cluster	1.27	2.20	0.58	0.53	.610	0.02

a. Greenhouse-Geisser corrected ANOVA

Table S5

Test statistics for post-hoc paired t-tests regarding ERP amplitudes in the PC and the VR condition for each cluster for the N1 time window (165-205ms).

	Paired Samples Statistics		*Paired Samples Test*					
			Paired Differences					
	M	*SD*	*M*	*SD*	*t*	*df*	*p*	*Cohen's d*[a]
PC: frontal	-260	1.85	-0.70	1.81	-1.92	24	.066	-0.38
VR: frontal	-1.91	2.04						
PC: mid-frontal	-1.75	1.45	-1.01	1.12	-4.50	24	<.001	-0.90
VR: mid-frontal	-0.74	1.38						
PC: left temporal	-0.59	1.10	-0.53	1.25	-2.13	24	.044	-0.43
VR: left temporal	-0.06	1.10						
PC: right temporal	-0.74	0.93	0.05	0.65	0.38	24	.705	0.08
VR: right temporal	-0.79	0.73						
PC: posterior	4.38	2.85	1.89	2.03	4.66	24	<.001	0.93
VR: posterior	2.48	2.48						
PC: centro-parietal	-0.35	0.98	-0.14	0.84	-0.83	24	.413	-0.17
VR: centro-parietal	-0.21	0.73						

a. Cohen's d uses the sample standard deviation of the mean difference.

Table S6

Test statistics for rmANOVA with the factors Modality, Repetition and Cluster for the L1 time window (220-800ms).

Tests of Within-Subjects Effects[a]

	Type III Sum of Squares	*df*	*Mean Square*	*F*	*p*	η^2
Modality	0.18	1.00	0.18	0.49	.490	0.02
Repetition	0.21	1.00	0.21	2.85	.104	0.11
Cluster	4165.47	1.76	2369.67	113.26	<.001	0.83
Modality*Repetition	0.02	1.00	0.02	0.45	.510	0.02
Modality*Cluster	86.55	1.63	53.02	7.02	.004	0.23
Repetition*Cluster	51.34	2.46	20.84	22.29	<.001	0.48
Modality*Repetition*Cluster	3.51	2.53	1.39	2.16	.112	0.08

a. Greenhouse-Geisser corrected ANOVA

Table S7

Test statistics for post-hoc paired t-tests regarding ERP amplitudes in the PC and the VR condition for each cluster for the L1 time window (220-800ms).

	Paired Samples Statistics		Paired Samples Test					
			Paired Differences					
	M	*SD*	*M*	*SD*	*t*	*df*	*p*	Cohen's *d*[a]
PC: frontal	-3.89	1.51	0.41	2.40	0.85	24	.406	0.17
VR: frontal	-4.29	2.42						
PC: mid-frontal	-2.92	1.24	-0.69	1.10	-3.13	24	.005	-0.63
VR: mid-frontal	-2.23	1.11						
PC: left temporal	-0.56	1.01	-0.84	0.82	-5.12	24	<.001	-1.02
VR: left temporal	0.28	0.68						
PC: right temporal	0.16	0.76	-0.26	0.84	-1.57	24	.130	-0.31
VR: right temporal	0.42	0.55						
PC: posterior	5.06	2.40	1.41	1.91	3.70	24	.001	0.74
VR: posterior	3.65	2.09						
PC: centro-parietal	-0.09	1.08	-0.23	0.82	-1.42	24	.169	-0.28
VR: centro-parietal	0.14	0.80						

a. Cohen's d uses the sample standard deviation of the mean difference.

Table S8

Test statistics for post-hoc paired t-tests regarding the RS effect for each cluster for the L1 time window (220-800ms).

	Paired Samples Statistics		*Paired Samples Test*					
			Paired Differences					
	M	*SD*	*M*	*SD*	*t*	*df*	*p*	*Cohen's d*[a]
First frontal	-4.39	1.69	-0.59	0.88	-3.35	24	.003	-0.67
Second frontal	-3.80	1.66						
First mid-frontal	-2.87	1.08	-0.60	0.54	-5.55	24	<.001	-1.11
Second mid-frontal	-2.27	1.06						
First left temporal	<0.01	0.87	0.28	0.64	2.22	24	.036	0.44
Second left temporal	-0.28	0.77						
First right temporal	0.27	0.60	-0.05	0.48	-0.50	24	.623	-0.10
Second right temporal	0.31	0.52						
First posterior	4.89	2.08	1.07	0.75	7.14	24	<.001	1.43
Second posterior	3.82	2.07						
First centro-parietal	-0.15	0.89	-0.35	0.34	-5.20	24	.001	-1.04
Second centro-parietal	0.20	0.85						

a. Cohen's d uses the sample standard deviation of the mean difference.

Table S9

Test statistics for rmANOVA with the factors Modality, Repetition and Cluster for the L2 time window (800-1500ms).

Tests of Within-Subjects Effects[a]

	Type III Sum of Squares	*df*	*Mean Square*	*F*	*p*	η^2
Modality	0.89	1.00	0.89	4.38	.047	0.15
Repetition	0.22	1.00	0.22	2.90	.102	0.11
Cluster	173.62	1.82	95.30	7.88	.002	0.25
Modality*Repetition	0.13	1.00	0.13	2.61	.119	0.10
Modality*Cluster	67.64	1.71	39.61	6.75	.004	0.22
Repetition*Cluster	5.26	2.54	2.07	2.12	.117	0.08
Modality*Repetition*Cluster	14.00	2.66	5.27	5.78	.002	0.19

a. Greenhouse-Geisser corrected ANOVA

Table S10

Test statistics for post-hoc paired t-tests regarding ERP amplitudes in the PC and the VR condition for each cluster for the L2 time window (800-1500ms).

	Paired Samples Statistics		*Paired Samples Test*					
			Paired Differences					
	M	*SD*	*M*	*SD*	*t*	*df*	*p*	*Cohen's d*[a]
PC: frontal	-0.71	0.85	0.56	2.10	1.33	24	.196	0.27
VR: frontal	-1.27	2.40						
PC: mid-frontal	-0.49	0.77	-0.26	0.98	-1.33	24	.195	-0.27
VR: mid-frontal	-0.22	1.10						
PC: left temporal	0.02	0.79	-1.09	0.88	-6.16	24	<.001	-1.23
VR: left temporal	1.10	0.74						
PC: right temporal	0.13	0.63	-0.62	0.79	-3.94	24	.001	-0.79
VR: right temporal	0.75	0.55						
PC: posterior	0.79	1.45	0.89	1.73	2.57	24	.017	0.51
VR: posterior	-0.10	1.78						
PC: centro-parietal	-0.04	0.73	0.06	0.68	0.46	24	.650	0.09
VR: centro-parietal	-0.10	0.62						

a. Cohen's d uses the sample standard deviation of the mean difference.

Table S11

Test statistics for post-hoc paired t-tests regarding the RS effect in the PC and the VR condition for each cluster for the L2 time window (800-1500ms).

	Paired Samples Statistics		*Paired Samples Test*					
			Paired Differences					
	M	*SD*	*M*	*SD*	*t*	*df*	*p*	*Cohen's d*[a]
PC_1: frontal	-0.61	0.87	0.20	1.18	0.85	24	.406	0.17
PC_2: frontal	-0.81	1.18						
VR_1: frontal	-1.75	2.55	-0.96	1.24	-3.85	24	.001	-0.77
VR_2: frontal	-0.79	2.40						
VR_1: frontal	-1.75	2.55	-1.14	2.32	-2.45	24	.022	-0.49
PC_1: frontal	-0.61	0.87						
VR_1: frontal	-1.75	2.55	-0.94	2.20	-2.13	24	.044	-0.43
PC_2: frontal	-0.81	1.18						
PC_1: mid-frontal	-0.38	0.82	0.21	0.74	1.42	24	.168	0.28
PC_2: mid-frontal	-0.59	0.89						
VR_1: mid-frontal	-0.39	1.20	-0.33	0.74	-2.17	24	.040	-0.43
VR_2: mid-frontal	-0.06	1.11						
PC_1: left temporal	0.02	1.04	0.01	1.05	0.06	24	.952	0.01
PC_2: left temporal	0.01	0.84						
VR_1: left temporal	1.26	0.91	0.32	0.91	1.75	24	.093	0.35
VR_2: left temporal	0.94	0.83						
PC_1: right temporal	-0.10	0.83	-0.46	0.86	-2.67	24	.013	-0.53
PC_2: right temporal	0.36	0.69						
VR_1: right temporal	0.82	0.71	0.14	0.70	0.99	24	.334	0.20
VR_2: right temporal	0.68	0.59						
PC_1: posterior	0.73	1.65	-0.11	1.03	-0.55	24	.590	-0.11
PC_2: posterior	0.84	1.42						
VR_1: posterior	0.06	1.96	0.32	1.09	1.48	24	.153	0.30
VR_2: posterior	-0.26	1.76						
PC_1: centro-parietal	0.01	0.80	0.10	0.62	0.77	24	.450	0.15
PC_2: centro-parietal	-0.09	0.78						
VR_1: centro-parietal	-0.06	0.65	0.09	0.46	0.98	24	.339	0.20
VR_2: centro-parietal	-0.15	0.67						

a. Cohen's d uses the sample standard deviation of the mean difference.

Table S12

Test statistics for post-hoc paired t-tests regarding the RS effects within the PC and the VR condition for the frontal, the midfrontal and the right temporal cluster for the L2 time window (800-1500ms).

	Paired Samples Statistics		*Paired Samples Test*					
			Paired Differences					
	M	*SD*	*M*	*SD*	*t*	*df*	*p*	*Cohen's d*[a]
PC: frontal difference	0.20	1.18	1.16	1.66	3.49	24	.002	0.70
VR: frontal difference	-0.96	1.24						
PC: mid-frontal difference	0.21	0.74	0.53	0.97	2.73	24	.012	0.55
VR: mid-frontal difference	-0.32	0.74						
PC: right temporal difference	-0.46	0.86	-0.60	1.18	-2.53	24	.018	-0.51
VR: right temporal difference	0.14	0.70						

a. Cohen's d uses the sample standard deviation of the mean difference.

Table S13

ERP components in source space.

	effect	center of gravity	MNI coordinates x	y	z	hemi-sphere	brain region	fig
P1	average	CG	-28	-98	-17	L	occipital pole	5.A1
			28	-98	-17	R	occipital pole	5.A1
	modality	CG	-72	-26	5	L	superior temporal gyrus	5.A2
N1	average		-28	-98	-17	L	occipital pole	5.B1
		CG	28	-98	-17	R	occipital pole	5.B1
	modality	CG	7	-105	-2	R	cuneus	5.B2
			-14	-69	-2	L	medial occipitotemporal gyrus	5.B2
L1	RS	CG	-50	-62	-17	L	lateral occipitotemporal gyrus	5.C1
			28	-76	-17	R	lateral occipitotemporal gyrus	5.C1
			28	-98	-17	R	occipital pole	5.C1
	modality	CG	-28	-98	-17	L	occipital pole	5.C2
			43	-69	-17	R	lateral occipitotemporal gyrus	5.C2
			28	-98	-17	R	occipital pole	5.C2
L2	VR RS	CG	-28	-98	-17	L	occipital pole	6.B
			28	-98	-17	R	occipital pole	6.B
			-65	-19	-24	L	middle temporal gyrus	6.B
	PC RS	CG	28	-98	-17	R	occipital pole	6.A
	RS difference	CG	-28	-98	-17	L	occipital pole	6.C
			-65	-11	-17	L	middle temporal gyrus	6.C
			50	-69	-17	R	inferior temporal gyrus	6.C

Note. CG = center of gravity, RS = repetition suppression effect.
